# Beyond Visual Inspection: A Systematic Review of Adjunctive Aids for the Early Detection of Oral Squamous Cell Carcinoma

**DOI:** 10.3390/jcm15062146

**Published:** 2026-03-11

**Authors:** Petra Claudia Camilla D’Orsi, Saman Warnakulasuriya, Francesco Perri, Luís Monteiro, Agostino Guida

**Affiliations:** 1 Scuola Superiore Meridionale, Via Mezzocannone 4, 80131 Naples, Italy; 2 King’s College London & WHO Collaboration Centre for Oral Cancer, London SE5 9RS, UK; saman.warne@kcl.ac.uk; 3 Head and Neck Medical Oncology Unit, Istituto Nazionale Tumori, IRCCS G. Pascale, 80131 Naples, Italy; 4 University Institute of Health Sciences (IUCS), Cooperativa de Ensino Superior Politécnico e Universitário (CESPU), 4585-116 Gandra, Portugal; 5 Unità Operativa Complessa Odontostomatologia Azienda Ospedaliera di Rilievo Nazionale “Antonio Cardarelli”, 80131 Naples, Italy

**Keywords:** mouth neoplasms, oral cancer, precancerous conditions, oral leukoplakia, oral lichen planus

## Abstract

**Background/Objectives**: The early detection of oral squamous cell carcinoma (OSCC), especially when in the presence of oral potentially malignant disorders (OPMDs), may be challenging and would assist in improving poor OSCC survival rates reported in the literature. We conducted a systematic review to evaluate the utility of adjunctive aids that could assist during clinical examination of the oral cavity to identify suspicious mucosal lesions. **Methods**: Three databases (CENTRAL, PubMed/MEDLINE, Embase) were screened, limiting results from 2015 to November 2025. Inclusion criteria were: articles written in English; investigating the diagnostic accuracy of diagnostic visual aids compared to conventional oral examination under white light in the assessment of oral mucosal lesions. Extracted data were analysed narratively. Studies not reporting diagnostic accuracy using biopsy results as the gold standard were excluded. **Results**: The search produced 137 articles; after removing duplicates, 105 were screened through inclusion/exclusion criteria, leading to 17 papers included in the review. Eight articles investigated diagnostic accuracy of narrow band imaging (NBI), seven visually enhanced lesion scopes (VELscopes), one Glasses for Oral Cancer Curing Light Exposed Screening (GOCCLES), one ViziLite chemiluminescence system, and two toluidine blue (TB). **Conclusions**: High study heterogeneity and lack of randomized clinical trials limit the conclusions of this review. In this context, among the investigated visual aids for expert use, NBI (sensitivity 85–100%, specificity 75–98%) emerges as the most promising tool (VELscope sensitivity 76–87.1%, specificity 21.4–90%; GOCCLES 66%, 48%; ViziLite 77.3%, 27.8%, TB 56.8–91%, 65.3–68%), due to its ability to highlight sub epithelial vascular abnormalities, considered as early indicators of dysplastic or neoplastic progression even. None of the investigated visual aids seem suited for screening purposes/use by the general dentist.

## 1. Introduction

Oral squamous cell carcinoma (OSCC) is a malignant neoplasm arising from the lining mucous membrane of the oral cavity [1]. OSCC represents the most common malignancy affecting the head and neck region [2], with an annual incidence rate of 389,485 new cases and 188,230 deaths/year [3]. The incidence rate for men is almost twice that of women, especially for patients between the 5th and 6th decade of life, with a large geographical variability [4].

Common risk factors include tobacco and excess alcohol consumption, with an approximately 38-fold increased risk of developing oral cancer for heavy smokers and heavy drinkers [5].

Additionally, ultraviolet and sun exposure is strongly associated with lip cancer development, most commonly affecting the lower lip. Other risk factors include immunosuppression, family history of OSCC, oral dysbiosis and, especially in some regions of the world, habit of chewing tobacco/areca nut [6]. The development of OSCC may be preceded by oral potentially malignant disorders (OPMDs) [7]. OPMDs are defined as a group of conditions with an increased risk of malignancy, with an estimated rate of transformation variable from 0.13% to 34% [7,8]. The clinical manifestations may vary, including white and/or red patches that configure patterns of leukoplakia and erythroplakia, respectively [9]. Other conditions include proliferative verrucous leukoplakia, oral submucosal fibrosis, oral lichen planus (OLP), and oral lichenoid lesions (OLLs) [10].

Although the oral cavity is easily accessible for clinical evaluation, up to 50% of oral cancers are diagnosed at an advanced stage (stage III and IV), needing invasive treatment and leading to poor prognosis. In fact, the overall 5-year survival rate remains low, approximately 60%, and such a poor overall rate is thus related with most cases being diagnosed at advanced stages. Diagnostic delay is partially due to absence of symptoms at the early stage [5]. Symptoms such as dysphagia, pain, and dysphonia are usually associated with deeper layers of invasion, which occur at advanced stages. For these reasons, if the diagnostic pathway begins at the start of the initial symptoms, prognosis is usually very good. Early diagnosis, which needs to occur when the patient is still asymptomatic or when the tumour is small in size, is thus the most effective step for reducing the invasiveness of treatments and improving patients’ quality of life and overall survival [11].

The American Dental Association (ADA) and the American Academy of Oral Medicine (AAOM) recommend opportunistic screening for OSCC and OPMDs during regular visit to the dentists, through conventional oral examination (COE) of oral mucosae [12,13]. On the other hand, systematic oral cancer screening programs of the general population are adopted only by few countries, due to the rarity of the disease and the lack of strong evidence of the efficacy of screening for OSCC in asymptomatic adults in primary care settings [14].

COE, which includes palpation and visualization of lesions under white light (WL), remains the primary method by which suspected OPMD/OSCC lesions are spotted. Detected lesions need to undergo biopsy and histological examination, which is still the gold standard for OPMD/OSCC diagnosis [5], as COE alone is largely dependent on the skill of the operator, and its ability to discriminate between benign or malignant conditions [15] is poor.

The gold standard for OPMDs/OSCC diagnosis (COE and biopsy with subsequent histological analysis) may also present limitations in applicability, due to poor patient compliance, especially in cases of chronic lesions, which require periodic biopsies.

In order to overcome such limitations, non-invasive chair-side diagnostic tools are being researched as clinical adjunctive techniques for the diagnosis and management of OPMDs/OSCC. This systematic review aims to evaluate the impact of emerging chair-side technologies for the diagnosis of OPMDs/OSCC.

## 2. Materials and Methods

This systematic review was conducted following the Preferred Reporting Items From Systematic Reviews and Meta-Analysis—PRISMA—2020 (Table S1) [16]. This systematic review was registered in PROSPERO with number 282032.

### 2.1. Search Strategy

A web search was performed on 13 January 2026, and the databases included were: Cochrane Central Register of Controlled Trials (CENTRAL), MEDLINE (via PubMed) and Embase. The search strategy included a combination of related free terms and MeSh terms in order to maximise sensitivity. The search query was:

mouth neoplasms [MeSH Terms] OR (“oral squamous cell carcinom*”[Title/Abstract]) OR (“oral cancer”[Title/Abstract]) OR (“oral potentially malignant disorder*”[Title/Abstract]) OR (“leukoplakia”[Title/Abstract]) (OR “erythroplakia”[Title/Abstract])

*AND*

“early diagnosis”[MeSH Terms] OR (“diagnos*”[Title/Abstract]) OR (“screening”[Title/Abstract]) AND “conventional oral examination” OR “white light” OR “visual inspection”.

Through search filtering, non-eligible publication types (see below) were excluded, and publication date was limited to the last 10 years. References were managed via Mendeley, and duplicates were removed.

### 2.2. Eligibility Criteria

Inclusion criteria were:

- English language;
- All types of descriptive and analytic studies (apart from those falling under the exclusion criteria) according to the Oxford Centre For Evidence Based Medicine (OCEBM) [17] regarding patients undergoing WL examination and examination with a visual aid prior to a biopsy with histopathological examination;
- Studies on humans.

Exclusion criteria were:

- Case reports and all type of reviews/meta-analyses;
- Articles not reporting diagnostic accuracy (true/false positive, true/false negative, positive predictive value—PPV, negative predictive value—NPV) using biopsy results as the gold standard;
- Articles not using WL as a control (e.g., comparing two visual aids).

Articles not meeting inclusion criteria and/or meeting exclusion criteria during any phase of the screening process were excluded.

### 2.3. Study Selection and Screening

Two authors (PCCD, AG) independently searched the literature according to the eligibility criteria, after which duplicates were removed. The screening was conducted in two stages: first, an independent review of all titles and abstracts, followed by a full-text evaluation of potentially relevant articles to confirm final inclusion. Screening evaluation and settling of disagreements between authors were performed by the senior author (SW).

### 2.4. Data Extraction

The data extracted from the selected articles was recorded in a standardized fashion using Microsoft Excel v 365. Scheduled data included information such as the first authors, journal published, the type of study, the study population, adjunctive visual tool analysed and the accuracy of test results.

### 2.5. Evaluation of Quality and Risk of Bias

Each article that was read in full was critically appraised for its methodology, quality and risk of bias using the “Quality Assessment of Diagnostic Accuracy Studies”—QUADAS-2 [18]. Through responding to a questionnaire, risk of bias was evaluated through assessment of 4 domains, patients selection, test in study, reference standard, flow and timing. For each study, the domain was classified as low, unclear and high risk of bias. Settling of potential disagreements between authors was performed by the senior author (SW).

## 3. Results

**Figure 1:**
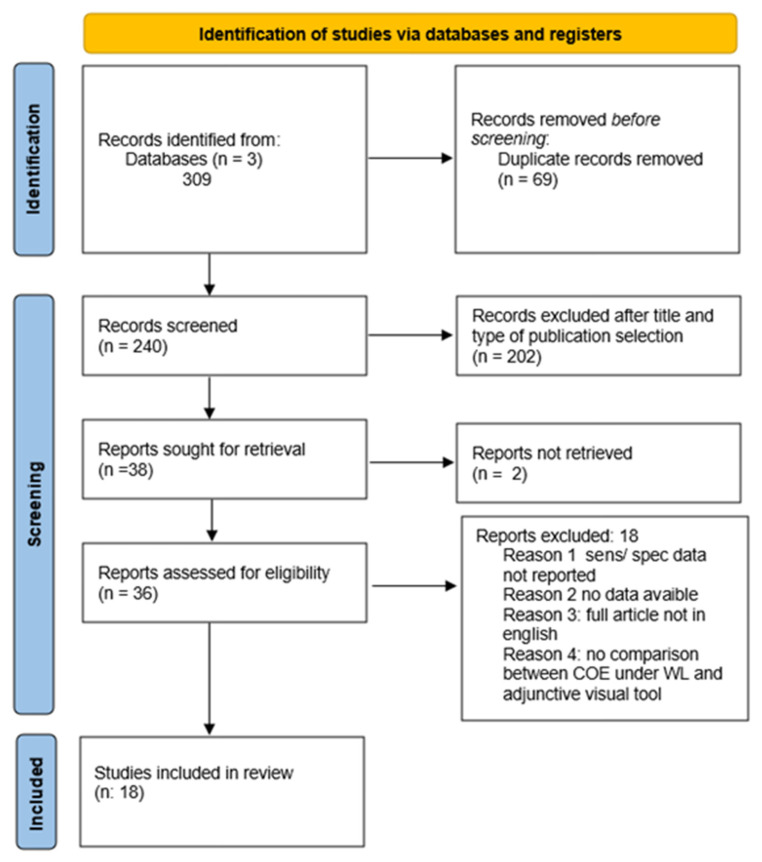
PRISMA flow diagram. Synthesis of the bibliographic analysis.

The study selection and screening process is summarised in Figure 1. Initial search strategy for articles investigating the diagnostic accuracy of visual diagnostic aids in OPMDs and OSCC detection compared to COE/WL yielded a total of 309 publications. Fifty-nine articles were retrieved from Cochrane Library, 109 from Embase and 141 from PubMed. After removing duplicates, 240 articles remained. After evaluating the titles and type of publication, 202 articles were discarded, and 38 were selected for full-text reading. Two articles were not retrieved as full text, and, at the end, 18 articles meeting the eligibility criteria/not meeting exclusion criteria were included in this review.

Among these, five articles are retrospective studies, six articles were prospective studies, four articles were cross-sectional studies, one article was a preliminary–descriptive study, one article was a comparative study, and one article was a randomized controlled trial/pilot study. The main characteristics and results of the included studies are summarised in Table 1.

**Figure 2:**
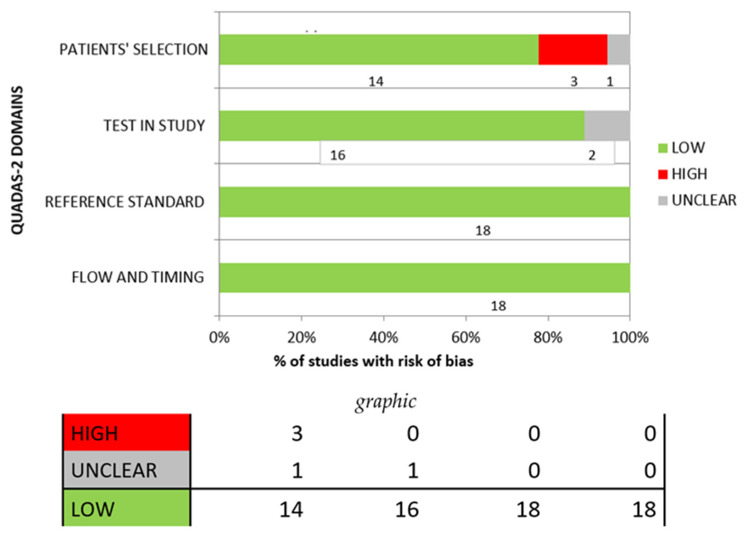
Overall QUADAS-2 risk of bias assessment.

Risk of bias QUADAS-2 assessment (summarised in Table 2, Figure 2) highlighted a low risk of bias for most publications.

Included publications testing effectiveness of visual aids could be grouped into four main categories: narrow band imaging (NBI), autofluorescence, chemiluminescence, and toluidine blue staining.

### 3.1. Narrow Band Imaging—NBI

**Figure 3:**
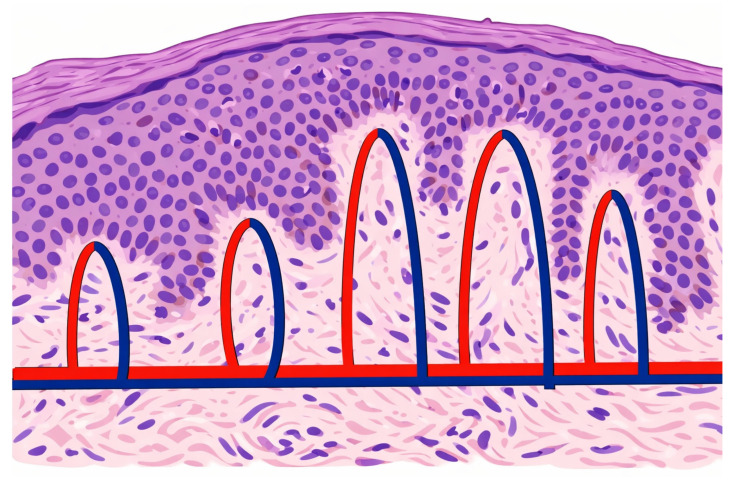
Intrapapillary capillary loops’ scheme, showing as NBI highlights the most superficial/close to the epithelium edge vascularisation. This image has been originally created by the authors with Adobe 2026 software.

NBI is a non-invasive endoscopic technique that enhances the visualization of microvascular changes using narrow band blue light (385–445 nm) and green light (510–570 nm), which are adsorbed by haemoglobin, highlighting the intrapapillary capillary loops (IPCLs) (see Figure 3) of the superficial layer of connective tissue [37].

Various IPCL classifications have been proposed in the scientific literature. The most used classification for the oral cavity was proposed by Takano et al. and modified by Xu et al., dividing IPCL into four types/12 subtypes [38,39]. From type I to IV, there are increasing chances of correlation with high grade dysplasia (HGD) and OSCC. Different studies have assessed the effectiveness of NBI in detection of early cancer in various mucosal systems, such as the larynx, stomach, oesophagus, and colon [40,41,42]. For the early detection of OPMDs and OSCC, NBI appears to have high sensitivity, specificity and accuracy compared to COE/WL.

Eight publications reporting the diagnostic accuracy of NBI in detecting OPMDs/OSCC were included in this review.

Vu et al. (2015) [20] conducted a prospective study on a total of 95 patients (85 with a previous diagnosis of OPMD, 10 new cases) who had at least one white, red or mixed white/red lesion on the oral mucosa. The diagnostic protocol involved inspection of the oral mucosa using COE, WL and NBI; the final diagnosis was made by biopsy and subsequent histopathological analysis.

The authors highlighted differences between COE and WL: both COE and WL clinical provisional diagnoses were associated with colour and border distinctness; WL enhanced visualization of 99.3% of lesions visualized by COE and aided the detection of 11 lesions missed by COE. On the other hand, NBI improved the visualization of 241 lesions detected by COE and 234 detected by WL and aided the detection of 24 lesions undetected by COE and 13 lesions missed by WL. Clinical values of sensitivity, specificity, PPV, NPV and accuracy for the detection of OPMDs (with histopathology as the gold standard) were for WL 40.54%, 78.57%, 55.56%, 66.67% and 63.44%, respectively; the same values for NBI were 43.24%, 75.00%, 53.33%, 66.67% and 62.37%, respectively. However, when the compared to COE/WL pre-biopsy, the sensitivity, specificity, PPV, NPV, and accuracy of the NBI were 100%, 74.63%, 92.38%, 100%, and 93.77%, respectively (when compared to COE), and 100%, 87.50%, 96.86%, 100%, and 97.43%, respectively (when compared to WL). In this study, NBI enhanced the visualization of lesions detected by COE and WL, aided the detection of lesions missed by COE and WL and correctly changed the clinical provisional diagnosis of one lesion, finally diagnosed as mild dysplasia by histopathology. Furthermore, the IPCL pattern was not visible for 45 of 217 lesions with keratosis, classified as IPCLs type 0.

Piazza et al. (2016) [21] performed a non-randomized prospective study on 128 patients never biopsied or previously treated for oropharyngeal cancer/oral cancer, with clinical diagnosis of leukoplakia/erythroplakia less than 3 cm in maximum diameter. The diagnostic protocol consisted of inspection using COE, WL and NBI, followed by surgical biopsy. After COE, lesions were divided into two groups (suspicious or innocuous), according to clinical presentation; histopathological reports were defined as positive in case of mild dysplasia to invasive carcinoma, while negative lesions included chronic mucositis, LP and keratosis. Based on the histological examination results, the sensitivity, specificity, PPV, NPV, and accuracy values for the three methods were 51%, 68%, 77%, 39%, and 56%, respectively, for COE; 78%, 73%, 86%, 61%, and 77%, respectively, for WL; and 89%, 85%, 93%, 78%, and 87%, respectively, for NBI. According to Youden’s index, the discrimination ability between suspicious lesions and innocuous ones was higher for NBI compared to COE (J: 0.74 vs. J: 0.19) and compared to WL (J: 0.74 vs. J: 0.51). Moreover, NBI had a false positive rate of 15% and a false negative rate of 11%. Furthermore, stratifying the cases for the type of epithelium and its thickness, no significant difference in terms of sensitivity, sensibility, PPV, NPV or accuracy was observed for NBI evaluation.

Contaldo et al. (2017) [22] performed a preliminary study by enrolling 31 consecutive patients with oral lesions of different origins, who underwent COE/WL examination, NBI examination and incisional/excisional biopsy. With regard to the NBI assessment, in order to define NBI positivity and to establish the cut off for malignancy, two groups of criteria were considered: the first group included lesions clinically considered as malignant and NBI-positive when IPCL patterns III–IV were detected; conversely, the second group included lesions clinically considered as malignant and NBI-positive when IPCL pattern IV was detected. The values of sensitivity, specificity, PPV and NPV of NBI-positive lesions for both IPCL types III–IV were 100%, 78%, 40%, and 100% respectively, while the values of sensitivity, specificity, PPV and NPV for NBI-positive lesions for IPCL type IV were 100%, 93%, 67%, and 100% respectively.

These data agree with those reported by Upadhyay et al. (2019) [26], who conducted a prospective cross-sectional study on 38 patients with suspected oral premalignant and/or malignant lesions, who subsequently underwent WLE, NBI examination and biopsy; the site of biopsy was chosen based on abnormalities of vascular loops from NBI assessment. Among 38 cases, 50% showed IPCL type I, 31% showed IPCL type II, and 19% showed IPCL type III; six out 38 cases showed no neovascularization under NBI evaluation. Values of sensitivity, specificity, PPV, and NPV of WL and NBI were 75.75%, 76.43%, 0%, 33.33% (for WL), respectively, and 93.93%, 80%, 3.125% and 66.66% (for NBI), respectively. The authors reported that, in finding malignant lesions, the diagnostic ability of NBI is superior compared to WL.

Guida et al. (2019) [27] performed a retrospective study on 149 consecutive patients who underwent both WL and NBI assessment; after 51 patients were discarded, the final cohort consisted of 98 patients and 106 oral lesions analysed, including patients with anamnesis of OLP. The authors reported that factors such as age, sex, smoking, use of dentures and anamnesis of OSCC were not associated with NBI IPCL patterns.

Moreover, IPCL type IV is strongly associated with OSCC/Cis diagnosis: when NBI revealed an IPCL type IV, histopathological analysis revealed OSCC/Cis/HGD in 91.7% of cases and low grade dysplasia (LGD) in 8.3% of cases; conversely, NBI IPCL type III revealed OSCC/Cis/HGD in 12.5% of total cases and 27.2% of cases when OLP patients were excluded. Particularly, values of sensitivity, specificity, PPV, NPV and accuracy for NBI IPCL types III–IV were 96.2%, 71.3%, 52.1%, 98.3% and 77.4%, respectively, when patients affected by OLP were included in statistical analysis; values of sensitivity, specificity, PPV, NPV and accuracy for NBI IPCL types III–IV were 96.2%, 80%, 71.4%, 97.6% and 85.5%, respectively, when OLP patients were excluded. The same statistical analysis was conducted by stratifying for solely NBI IPCL type IV; values of sensitivity, specificity, PPV, NPV and accuracy were 84.6%, 96.0%, 91.7%, 92.3% and 92.1%, respectively, when OLP patients were included in the assessment, while these values were 84.6%, 96.0%, 91.7%, 92.3%, and 92.1% when OLP patients were excluded. The authors concluded that the presence of OLP patients in the final cohort did not influence NBI reliability in detecting lesions positive for OSCC/Cis/HGD.

In a further study, Guida et al. (2021) [30] conducted a multicentric retrospective study on 84 patients affected by oral lesions from OLP, OLL and oral lichenoid reactions (OLRs). Patients had undergone both WL and NBI inspection—performed by two reviewers independently—and finally surgical biopsy (note that the choice of site of biopsy was guided only by the WL examination). The concordance between two reviewers was examined with a Fleiss kappa value of 0.76%. OLP–OLL–OLR were related to types I–II of IPCLs. Conversely, lesions showing IPCL types III–IV were related to diagnosis of OSCC/Cis with values of sensitivity, specificity, PPV and NPV of 100%, 92.6%, 33.3% and 100%, respectively. The authors highlighted that, when only IPCL type IV was considered as a positive outcome, these values all reached 100%. The study has shown high sensitivity for NPV in detecting OSCC when IPCL types III–IV were found, while it showed high specificity and PPV in detecting OSCC when only IPCL type IV was considered as a positive outcome. The authors concluded that IPCL type IV could be a clinical marker of malignancy development in patients undergoing long-term follow up for OLP–OLL–OLR.

Deganello et al. (2021) [33] performed a prospective study on 56 patients with anamnesis of biopsy-proven OLP. Patients were evaluated by three examiners independently, and the interobserver reliability was assessed using pairwise Cohen’s kappa. The site of biopsy was chosen, focusing on the worse NBI IPCL type found. Macroscopic appearance included leukoplakia in 77% of cases and erythro-leukoplakia in 16% of cases, while 7% of lesions showed an erosive feature. Biopsy was classified as positive in the case of histopathological diagnosis of HGD/OSCC, and negative in the case of OLP without dysplasia or with LGD. Considering histopathology as the gold standard, values of sensitivity, specificity, PPV, NPV and accuracy of both WL and NBI were 80% vs. 100%, 94% vs. 98%, 80% vs. 100%, 57% vs. 71% and 78% vs. 96%, respectively. The authors concluded that the application of NBI had a high efficacy in the evaluation of lesions arising in OLP.

Similarly, Nair et al. (2021) [32] performed a prospective study on 50 patients naïve with 51 visible and accessible lesions of oral mucosa. All patients underwent surgical biopsy to determine the histological diagnosis. Values of sensitivity, specificity, PPV, NPV and accuracy of WL compared to histopathology were of 74.07%, 79,17%, 73.8%, 80.0% and 76.47%, respectively. NBI showed values of sensitivity, specificity, PPV, NPV and accuracy of 92.67%, 90.16%, 92.56%, 91.67% and 92.16%, respectively. The Cohen’s kappa value of interobserver agreement was 0.881%; this result is related to a high reliability and a high reproducibility of NBI assessment as a diagnostic tool for oral cavity lesions.

Ota et al. (2022) [34] conducted a single-centre, non-interventional cross-sectional study on 60 patients whose lesions involved the oral mucosa suspicious for OPMDs/OSCC with no previous treatment. Patients meeting the inclusion criteria subsequently underwent COE/WL and NBI assessment: finally, a surgical biopsy was performed. Ten healthy volunteers were also involved in the study; the same subsites of the oral mucosa were screened, but no biopsy was performed on volunteers. Lesions were assessed by NBI by two examiners: intra- and interobserver reliability was assessed using Cohen’s kappa value (measuring 0.75% and 0.81%, respectively). At the end, following histopathological analysis, the sample included, excluding the 10 volunteers, 16 OLP, 31 leukoplakia and 13 OSCC. Interestingly, there was a significant difference in assessing the extent of OLP/leukoplakia lesions between COE/WL and NBI, with an increased extent of lesions highlighted by NBI; on the other hand, there were no differences between COE/WL and NBI in assessing the extent of OSCC lesions. Under NBI assessment, IPCL type distribution varied based on the disease: OSCC lesions showed clearly advanced IPCLs, with types III–IV strongly associated with malignant transformation. The diagnostic accuracy of IPCL type III–IV to determine the possibility of OSCC was assessed at 85%. Values of sensitivity, specificity, PPV and NPV were 100%, 80.9%, 59.1% and 100%, respectively.

Finally, Debnath et al. (2024) [36] performed a study on 80 patients, among whom 68 showed lesions suggestive of malignancy. Thirty-three lesions affected the oral mucosa, 16 were located in oropharynx, 13 involved the hypopharynx, and six affected the larynx. The diagnostic protocol included inspection under WL, NBI and surgical biopsy. The detection rates of WL and NBI, depending on the lesions’ location, were 30% vs. 100% for oral cavity lesions; for oropharynx lesions these values were 69% vs. 81%; for lesions located in hypopharynx the diagnostic rates were 38% vs. 100%, while for larynx’s lesions the diagnostic rates were 0% and 83%, respectively (such values have been in all likelihood influenced by the fact that only six larynxes were evaluated). The diagnostic rate was assessed at 38% for WL and at 95% for NBI. Overall, values of sensitivity, specificity and accuracy for detecting malignant lesions for both WL and NBI were 82.9% vs. 100%, 94.74% vs. 94.74% and 85.92% vs. 98.59%, respectively.

### 3.2. Autofluorescence

Autofluorescence exploits the presence within the oral mucosa of molecules called fluorophores, which, when excited by light of a certain wavelength, emit energy. These are endogenous molecules, including structural proteins such as collagen and elastin, cofactors such as Nicotinamide Adenine Dinucleotide (NADH) and Flavine Adenine Dinucleotide (FAD), and other amino acids. In normal tissue, when excited by light with a wavelength between 370 and 460 nm, these molecules emit green areas. On the contrary, in dysplastic or frankly neoplastic tissues, they show dark areas on autofluorescence, a condition known as loss of fluorescence (LAF) or fluorescence visualization loss (FVL). This is due to structural alterations caused by epithelial dysplastic changes or neoplastic transformation, including:

- Break-up of the collagen cross-links;
- Increase in blood supply due to micro-vascularization and inflammation;
- Reduction in FAD and NADH [29,43].

There are several devices that utilise these tissue properties, but the most widely used is the visually enhanced lesion scope (VELscope), which uses tissue autofluorescence with a wavelength of blue light (between 400 and 460 nm) to enhance the visibility of oral submucosal abnormalities. Based on VELscope visualization, three fluorescence phenomena can be observed: FVL, typical of dysplastic/neoplastic tissue; fluorescence visualization retained (FVR), typical of normal mucosa; and fluorescence visualization increase (FVI), generally associated with increased keratosis or plaque deposition [19].

In this review, eight studies investigating the diagnostic accuracy of VELscope vs. COE are presented. One article evaluates the diagnostic accuracy of the EVINCE autofluorescence system vs. COE, and one article investigates the diagnostic performance of the Horus UOC 100™ digital autofluorescence camera in detection of OPMDs and OC.

One article evaluates the accuracy of Glasses for Oral Cancer Curing Light Exposed Screening (GOCCLES) in the evaluation of suspicious oral lesions. GOCCLES is a medical device using an optical filter consisting of a three-layer laminal optical structure that allows isolation of the fluorescent component emanating from the FAD (515 nm). Similarly to VELscope, lesions are classified into three categories (FVL, FVI, FVR) based on fluorescence phenomena.

Awan et al. (2015) [19] performed a prospective study on 126 patients with white, red and mixed white/red lesions diagnosed as OPMDs. Diagnostic protocol included COE and evaluation of lesions using three adjunctive tools (VELscope, ViziLite, Tblue) by two independent calibrated examiners; then, a surgical biopsy was performed in order to determine presence of dysplasia or carcinoma. Clinically, lesions were assessed as leuko-erythroplakia (70), frictional keratosis (13), OLP/OLR (32), chronic hyperplastic candidiasis (9), or oral submucosis (two).

Out of 70 cases classified as leuko/erythroplakia, under VELscope examination, 61 showed FVL, while nine showed FVR; interestingly, all nine cases of erythroplakia showed FVL. Values of sensitivity and specificity of VELscope compared to COE/WL and biopsy were 87.1% and 21.4%, respectively. Other OPMDs, including OLR/OLP, chronic hyperplastic candidiasis (CHC) and oral submucous fibrosis (OSF), under VELscope assessment showed different autofluorescence phenomena: out of 32 OLP/OLR, 90.6% of cases showed FVL; out of nine CHC, 5% of cases showed FVL; and out of two OSF, 100% of cases showed FVL. Among lesions diagnosed as dysplasia under histopathology, 84% of cases showed FVL. Interestingly, 100% of lesions with severe dysplasia showed FVL. Values of sensitivity, specificity, PPV and NPV in distinguishing lesions suspicious for HGD were 84.1%, 15.3%, 37.8% and 61.1%, respectively. The authors concluded that autofluorescence is able to identify most oral mucosal changes but not specifically oral leuko/erythroplakia nor epithelial dysplasia.

Ganga et al. (2017) [24] performed a prospective study on 200 oral mucosal lesions that subsequently underwent WL and VELscope examination; a biopsy was also performed, and the histopathological result was used as the gold standard. For histopathology, 175 out 200 lesions were benign, and 25 were positive for malignancy. Among these, 19 lesions showed FVL under VELscope. Comparing VELscope assessment to histopathology, values of sensitivity, specificity, PPV, NPV were 76%, 66.29%, 24.36% and 95.08%, respectively.

Leuci et al. (2020) [28] made a cross-sectional pilot study on 35 patients affected by previously diagnosed OPMDs, who subsequently underwent COE/WL and VELscope examination performed by two general dentists with different levels of experience. Based on the clinical aspect under COE, lesions were classified as benign or suspicious for malignancy; then, VELscope examination was performed, and at the end lesions underwent surgical biopsy with excision margins guided by VELscope images. The gold standard was the biopsy result. Results showed different values of sensitivity, specificity, PPV and NPV between COE and VELscope, with different outcomes depending on the examiner’s experience. VELscope showed sensitivity, specificity, PPV and NPV of 53.3%, 70%, 57.1% and 66.7%, respectively, as reported by the unskilled dentist; sensitivity, specificity, PPV and NPV were 86.7%, 90%, 86.7% and 90%, respectively, reported by the skilled operator. The COE, when performed by the skilled examiner, showed values of sensitivity, specificity, PPV and NPV of 73.3%, 65%, 61.1% and 76.5, respectively.

These results are concordant with those reported by Jabbar et al. (2020) [29] in a cross-sectional study on 50 patients who underwent COE/WL, VELscope examination and biopsy. Clinically, oral lesions mostly presented as white patches (50%), followed by red patches (24%) and ulcerate and exophytic lesions (14%). Histopathology showed benign results in 74% of cases, dysplasia in 14% of cases and OSCC in 12%. Among benign lesions, AF showed FVL in 35% of cases; conversely, among dysplastic lesions, AF showed FVL in 86% of cases; OSCC lesions showed FVL in 67% of cases. Values of sensitivity, specificity, PPV and NPV were 76.92%, 64.86%, 43.48% and 88.88%, respectively. The authors concluded that low specificity values reflect VELscope’s weakness in distinguishing high-risk lesions from low-risk ones.

Giovannacci et al. (2021) [31] conducted a prospective study on 108 lesions from 60 patients with a clinical diagnosis of OPMDs/OSCC that subsequently underwent COE/WL, VELscope, and biopsy. For each lesion’s parameters, such as appearance of lesion (white, red or mixed), histological diagnosis and clinicopathological diagnosis were assessed. Among white lesions, 66% of cases showed FVI, 29.8% of cases showed FVR, and 4.3% of cases showed FVL. Among white/red lesions, 50% of them showed FVL, 27.5% of cases showed FVI, and 22.5% of cases were normo-fluorescent. By considering the degree of AF in a binary way (normal or altered), the percentage of cases in which the AF was altered increased with an increase of invasiveness of the disease. AF was abnormal in 36% of cases with no dysplasia, in 75.9% of cases with mild–moderate dysplasia, and in 100% of cases of OSCC in situ or invasive and verrucous carcinoma. Particularly, lesions diagnosed as OSCC showed FVL in 73.3% of cases, while lesions diagnosed as verrucous carcinoma showed FVI in 87.5% of cases; in cases with no dysplasia or with mild–moderate dysplasia, AF mostly showed FVI. Predictive values of sensitivity, specificity and accuracy of AF phenomena related to the “group histopathological diagnosis” were 81.0%, 76.7% and 78.8%, respectively.

Chiang et al. (2019) [25] performed a comparative study on 126 patients suspected of having oral mucosal disorders who subsequently underwent COE/WL, autofluorescence assessment and biopsy. In this study, autofluorescence evaluation was made using a Horus UOC 100 digital autofluorescence camera. After COE, 68 patients were diagnosed as having an OPMD, 10 patients were diagnosed as having OSCC, and 48 patients were diagnosed as having other non-OPMD conditions. After histopathological assessment, 63 patients were confirmed to have epithelial dysplasia, OSCC diagnosis was confirmed in six cases, while 57 lesions were categorized as non-dysplastic. Among 68 cases diagnosed as OPMDs, AF assessment showed FVL in 53 cases, while among 63 cases confirmed to have epithelial dysplasia, AF showed FVL in 56 cases. Autofluorescence showed values of sensitivity, specificity, PPV, NPV and accuracy of 77.94%, 35.42%, 63.10%, 53.13% and 60.34%, respectively, when all OPMD cases were considered; when considering epithelial dysplasia cases, values of sensitivity specificity, PPV, NPV and accuracy were 88.89%, 43.86%, 63.34%, 78.13% and 67.50%, respectively. Finally, stratifying for only dysplasia cases among OPMDs, values of sensitivity, specificity, PPV, NPV and accuracy were 87.50%, 72.73%, 94.23, 53.33 and 85.07%, respectively.

Simonato et al. (2016) [23] conducted a pilot study on 15 patients with oral mucosal lesions who were randomly selected and who subsequently underwent COE/WL and AF assessment using the EVINCE device, performed by two examiners with different levels of skill, independently; histopathological analysis was used as the gold standard to measure diagnostic accuracy. Results showed different values of sensitivity, specificity, PPV, NPV and accuracy between COE and fluorescence visualization for both examiners. AF showed values of sensitivity, specificity, PPV, NPV and accuracy of 100%, 46.15%, 22.22%, 100% and 53.33%, respectively, for the unskilled examiner in diagnosing epithelial dysplasia; interestingly, analogous values were obtained when AF assessment was performed by an expert examiner. Values of sensitivity, specificity, PPV, NPV and accuracy of AF assessment in diagnosing OPMDs were 100%, 60%, 55.55%, 100% and 73.33%, respectively, for both examiners. This study suggests a possible application of AF visualization to improve general dentists’ ability to identify suspicious oral lesions and refer patients to specialized care centres.

Regarding diagnostic performance of GOCCLES, Lajolo et al. (2022) [35] performed a retrospective study on 25 patients and 42 lesions with a clinical diagnosis of OPMDs needing biopsy (made on 41 lesions). Patients first underwent COE/WL, AF examination with GOCCLES and inspection with Tblue. After COE/WL, lesions were clinically classified as OLL/OLP/OLR, erythroplakia, leukoplakia or mixed and verrucous leukoplakia. After GOCCLES examination, 18 out of 42 lesions showed FVR, 23 lesions showed FVL, but only eight cases were positive with histology (true positive), while 15 samples showed FVL but had a negative histology. Values of sensitivity, specificity, PPV, NPV and accuracy were 66%, 48%, 34%, 77% and 53%, respectively.

### 3.3. Chemiluminescence

One article investigates the effectiveness of a chemiluminescence system called ViziLite in comparison with COE/WL in the early detection of OPMDs/OSCC.

The ViziLite system begins with a 1% acetic acid oral rinse used for one minute to remove surface glycoproteins and lightly dry the oral mucosa. After this, a chemiluminescent blue-white light, emitting wavelengths between 490 and 510 nm, is activated to inspect the tissues. Under this light, normal tissues absorb the illumination and appear blue, while abnormal cells—with their higher nuclear-to-cytoplasmic ratio—reflect more light and show up as brighter, well-defined “aceto-white” areas [44].

Awan et al. (2015), [19] in their prospective study on 126 patients, examined the diagnostic performance of ViziLite. Among 70 lesions clinically classified as leuko/erythroplakia, 54 showed aceto-whitening (positive test), while 16 showed a negative test. Among other OPMDs (OLP/OLL/OLR, CHC and OSF), out of 32 lesions assessed as OLR/OLP, 75% of cases were aceto-white under ViziLite; out of nine CHC, 77.7% of cases were positive under ViziLite; and among two cases of OSF, 50% showed positivity under ViziLite. Regarding lesions diagnosed as dysplastic under histopathology, 77.3% of cases showed aceto-whitening. Values of sensitivity, specificity, PPV, and NPV for ViziLite were 77.3%, 27.8%, 39.5% and 66.7% respectively.

### 3.4. Vital Staining

The most common vital staining used in the detection of OPMDs/OSCC is the toluidine blue test (Tblue). Tblue is a cationic metachromatic dye that may selectively bind to free anionic groups such as sulphate, phosphate, and carboxylate radicals of large molecules, including phosphate groups of nucleic acids. The absorption of toluidine blue by dysplastic/neoplastic tissues could be due to molecular aberrations, including loss of heterozygosity (LOH) and loss of tumour suppressors. It is postulated that the increased amount of DNA and RNA in neoplastic cells compared to normal epithelial cells is responsible for uptake of the stain by malignant cells [45].

Based on the colour intensity of tissue evaluated under Tblue staining, it is possible to highlight two different settings: lesions showing no coloration or pale royal blue are classified as negative, while lesions showing coloration as dark royal blue are classified as positive [35].

Two studies meeting the inclusion criteria evaluating the diagnostic accuracy of Tblue have been included in this review.

Awan et al. (2015) [19] analysed diagnostic performance of vital staining (Tblue) in distinguishing between high-risk lesions and low-risk lesions. Among lesions clinically classified as leukoplakia/erythroplakia, 37 cases showed positive test, including seven out of nine cases of erythroplakia. Out of 32 cases of OLL/OLP/OLR, 21.8% showed positivity with Tblue, 5% of CHC showed positivity with Tblue, while 100% of OSF were positive with Tblue examination. Among lesions with histological diagnosis of dysplasia, Tblue was positive in 25 cases out of 44; sensitivity for detecting high-risk dysplasia amounted to 85.7%, whereas sensitivity for detecting low-risk dysplasia was 48.3%. Overall, values of sensitivity, specificity, PPV and NPV were 56.8%, 65.3%, 50.0% and 71.2%, respectively. Tblue’s sensitivity in detecting dysplasia cases was 56.8%, but when lesions were grouped into high-risk dysplasia and low-risk dysplasia, sensitivity rose to 85%.

Lajolo et al. (2022) [35] evaluated the diagnostic performance of Tblue in detecting and distinguishing OPMDs. Among 41 lesions, 20 were classified as dark royal blue (positive), while 21 were classified as pale royal blue (negative). Eleven out of 20 lesions were positive under histology, while the remaining nine positive lesions were assessed as OLL (two cases), erythroplakia (four cases) and leukoplakia (one case). Among 21 lesions showing pale royal blue under Tblue, 20 were true negatives cases under histology, while one case had a histology of verrucous proliferative carcinoma. Values of sensitivity, specificity, PPV, NPV and accuracy were 91%, 68%, 55%, 95% and 5%, respectively. In Table 1 we list a summary of the results described above.

## 4. Discussion

In the past years, various visual and vital staining techniques have been proposed as diagnostic aids to COE and WL for evaluation of oral lesions, especially for those suspicious of malignancy. Although conventional clinical inspection is considered the first -line assessment, there are some limitations, primarily related to the experience of the operator performing it. The classic presentation of an OPMD or carcinoma in situ as white or red lesions, with or without ulceration, can cause difficulties in differential diagnosis with other benign conditions. Currently, however, the definitive diagnosis of OPMDs and/or OSCC is based on biopsy (incisional or excisional, depending on the size of the lesion) and subsequent histopathological analysis. However, biopsy is an invasive procedure, often poorly tolerated by patients, and cannot be administered on a large scale for the evaluation of all oral mucosal lesions, especially for screening or for the follow up of chronic OPMD conditions. Furthermore, difficulties are often encountered in selecting the site for biopsy sampling, leading to under-diagnosis or misdiagnosis [46].

Adjunctive visual aids, such as NBI, autofluorescence, chemiluminescence, and vital staining alone or in combination, have gained recognition in the assessment of oral lesions suspicious for malignancy, supporting conventional clinical inspection and white light examination [47,48]. These techniques could highlight features of lesions missed by COE/WL, enhancing a clinician’s ability to identify OPMDs at high-risk of transformation.

When analysing the effectiveness of different visual aids, as emerged from this systematic review, high heterogeneity may limit a conclusion on evidence per a single visual aid and comparatively between the different devices. Despite the analysed lesion spectrum being similar between the studies (OPMDs and initial OSCCs), the setting is different in most papers; NBI is usually used by highly-trained second-level hospital physicians, while the other visual aids are intended for general use (especially the VELscope). This possibly levelled NBI values to the highest results. As visual aids do not have quantitative thresholds but show an image, which need interpretation, the expertise of the examiner is possibly the key influencing factor of the results. Another theoretical limitation for visual aids that are based on electronic devices (NBI, VELscope, GOCCLES, ViziLite) may be variations between software/hardware versions, which have never been assessed in the literature per each device.

With all the aforementioned limitations, among the additional visual aids investigated, NBI emerges as the most promising tool for second-level centres, due to its ability to highlight subepithelial vascular abnormalities, considered as early indicators of dysplastic or neoplastic progression, when used by trained physicians. The diagnostic accuracy of NBI, based on the results obtained from the analysis of the studies included in this review, confirms high sensitivity values ranging from 85% to 100% and excellent specificity values ranging from 75% to 98%. Considering IPCL type III–IV patterns as predictors of HGD/Cis/OSCC, diagnostic accuracy achieves values between 90% and 100%. Particularly, IPCL type IV is associated with sensitivity values in the detection of HGD/Cis/OSCC that reach almost 100%. According to our results, NBI is also associated with excellent PPV and NPV values, specifically, NPV values ranging from 78% to 100%. The low percentage of false negatives makes NBI an excellent additional device in the assessment of chronic oral mucosal lesions undergoing long-term follow up. Specifically, considering NPV values reaching 100%, many unnecessary biopsies could be avoided, and a less-intensive follow-up could be performed, in the absence of atypical NBI IPCL types [33]. Moreover, patients affected by OLP/OLL/OLR could be followed up with NBI assessment, performing a new biopsy when IPCL type IV is revealed [27]. In fact, IPCL type IV is strongly associated with HGD/Cis/OSCC development, possibly being a clinical marker of malignant transformation, with a sensitivity of almost 100% [22,30]. Additionally, all studies included in this review show a high reliability and high reproducibility of NBI assessment when performed by two or more examiners independently, with an overall Cohen’s kappa value of 0.80%. Therefore, it can be asserted that NBI is a reliable diagnostic aid in assessing the progression of oral mucosa lesions and in selecting a site for biopsy, and when choosing the area showing the worst IPCL type [22,34]. Interestingly, in all included studies, benign conditions were always associated with IPCL types I–II.

However, there are some limitations associated with the use of NBI. Currently, there is a lack of RCTs giving final evidence that NBI-assisted follow-up and biopsy would yield a superior result. Several studies included in this review reported the occurrence of IPCL type III even in some benign conditions, characterised by chronic inflammation and/or hypervascularisation [34], and this may represent a limitation for risk stratification. Furthermore, the evaluation of the vascular pattern can be influenced by characteristics of the epithelium, in particular by its thickness and the presence of keratin, as well as by the presence of foci of bleeding, which appear as dark spots under NBI light [22], possibly limiting lesion detection. Additionally, NBI is an expensive tool available only in some secondary care centres and it therefore cannot be recommended for screening procedures, despite its ease of use and excellent inter-operator reproducibility. Finally, such high values of inter-operator reproducibility may be influenced by the fact that NBI is usually used by highly trained and focused personnel in secondary care centres, while other visual aids have been assessed by general dentists.

On the other hand, diagnostic tools such as VELscope, GOCCLES, ViziLite and toluidine blue are less expensive and are also used in primary care centres, although they may not be easy to calibrate for use among general dentists. Particularly, VELscope assessment is characterised by a low inter- and intra-operator reproducibility; in fact, its diagnostic accuracy is significantly influenced by the experience of the operator performing the assessment, with sensitivity and specificity values varying largely between a skilled examiner and an unskilled one [28].

Moreover, regarding the overall diagnostic accuracy emerging from the analysis of the included articles, VELscope shows values of sensitivity and specificity ranging from 72% to 87% and from 65% to 77%, respectively. Similarly, GOCCLES exhibits an overall diagnostic accuracy of 53%, with low sensitivity and specificity values. Particularly, the low specificity reflects VELscope’s weakness in distinguishing high-risk lesions from low-risk ones [29]. Additionally, it has reported a high percentage of false positives, especially in lesions with high inflammatory levels, probably due to high subepithelial blood flow and altered metabolic activity of inflamed mucosa [24,28].

Similar results were found for ViziLite assessment, which shows a sensitivity of 77.3% in detecting oral mucosa patches, but a specificity of 27.8% in correctly distinguishing high-risk lesions.

Finally, Tblue shows an overall accuracy of 75%, with values of sensitivity ranging from 57% to 91%% and specificity ranging from 65% to 68% in assessing high-risk lesions. Interestingly, when VELscope, ViziLite and Tblue are used in combination, the highest specificity values are reported.

## 5. Conclusions

High heterogeneity between studies limits the conclusions of this systematic review. In a second-level centre used by trained physicians, NBI is associated with the best diagnostic efficacy, especially for follow-up of chronic high-risk oral mucosal lesions and for selecting the site for biopsy, showing high sensitivity and specificity. Still, even if studies from different centres gave similar results, highlighting a promising reproducibility after proper training, there is no RCT giving final evidence for such applications.

None of the devices analysed seem useful for oral cancer screening purposes in the general population, for which conventional oral examination inspection of the oral mucosa under white light represents a useful, low-cost and effective screening method. VELscope assessment is associated with a high inter- and intra-operator variability, but studies assess its applicability not only with trained dedicated physicians but with general dentists too, possibly influencing such results. Devices such as ViziLite and toluidine blue require time for execution, which makes them impractical for screening purposes.

All diagnostic support devices require a learning curve for interpreting the clinical results.

## Figures and Tables

**Table 1:** Main characteristics of articles included and the diagnostic accuracy of adjunctive tests.

Author	Journal and IF	Type of Study	Study Cohort	N° Patients	Adjunctive Visual Aid Investigated	Diagnostic Accuracy Compared to COE/WL
Awan K. et al., 2015 [19]	*Clinical Oral Investigations*	Retrospective study	Patients with white, red and mixed white and red lesions—ONLY OPMDS	126	VELscope, ViziLite and Tblue	Leukoplakia/Erythroplakia group Se: 84.1-77.3-56.8% Sp: 21.4-26.8 and 67.9% Dysplasia group Se: 84.1-77.3-56.8; Sp: 15.3-27.8-65.8%
Vu A. et al., 2015 [20]	*Oral Disease*	Prospective study	White, red and mixed white and red lesions of oral mucosa	95	NBI	Se: 43.24%; Sp: 75.00%; PPV: 53.33%; NPV: 66.67% Acc: 62.37% (when histology is the GS)
Piazza C. et al., 2016 [21]	*European Archives Of Oto-Rhino Laryngology—HN Surgery*	Prospective study	Leukoplakia and erytroplakia lesions less than 3 cm in diameter	128	NBI	Se: 89%; Sp: 85%; PPV: 93%; NPV: 78%; Acc: 87%
Contaldo M. et al., 2017 [22]	*Journal of Biological Regulators and Homeostatic Agents*	Preliminary–descriptive study	Oral lesions	31	NBI	Se: 100%; Sp: 93%; PPV: 67%; NPV:100%
Simonato L.E. et al., 2017 [23]	*Photodiagnosis and Photodynamic Therapy*	RCT/pilot study	OPMDs/dysplasia	15	autofluorescence	OPMDs—unskilled examiner: Se: 100%; Sp: 46.15%; PPV: 22.22%; NPV: 100%; Acc: 53.33%. OPMDs—skilled examiner: Se: 100%; Sp: 46%; PPV: 22.22%; NPV: 100%; Acc: 53.33% Dysplasia—unskilled examiner: Se: 100%; Sp: 60%; PPV: 55.55%; NPV: 100%; Acc: 73.33%. Dysplasia—skilled examiner: Se: 100%; Sp: 60%; PPV: 55.55%; NPV: 100%; Acc: 73.33%
Ganga et al., 2017 [24]	*Oral Oncology*	Prospective study	Lesions of oral mucosa	200	VELscope	Se: 76%; Sp: 66.29%; PPV: 24.36%; NPV: 95.08%
Chiang T.E. et al., 2019 [25]	*Clinical Oral Investigation*	Comparative study	Oral mucosa disorders	126	Autofluorescence	OPMDs: Se: 77.94%; Sp: 35.42%; PPV: 63.10%; NPV: 53.13%; Acc: 60.34%; Dysplasia: Se: 88.89%; Sp: 43.86%; PPV: 94.23%; NPV: 78.13%; Acc: 85.07%
Upadhyay A. et al., 2019 [26]	*Indian Journal of Otolaryngology and Head and Neck Surgery*	Prospective cross-sectional study	Suspicious lesion of oral cavity	38	NBI	Se: 93.93%; Sp: 80%; PPV: 3.125%; NPV: 66.66%
Guida et al., 2019 [27]	*BMC Oral Health*	Retrospective study	Oral cavity lesions	98	NBI	IPCLs pattern III–IV including OLP patients: Se: 96.2%; Sp: 71.3%; PPV: 52.1%; NPV: 998.3%; Acc: 77.4%. Not including OLP patients: Se: 96.2%; Sp: 80%; PPV: 71.4%; NPV: 97.6%; Acc: 85.5%. IPCLs pattern IV including OLP patients: Se: 84.6%; Sp: 97.5%; PPV: 91.7; NPV: 95.31%; Acc: 94.3%. Without OLP: Se: 84.6%; Sp: 96%; PPV: 91.7; NPV: 92.3; Acc: 92.1
Leuci et al., 2020 [28]	*JCM*	Cross-sectional pilot study	OPMDs	35	VELscope	SD Se: 73.3%; Sp: 65%; PPV: 61.1%; NPV: 76.515 UD: Se: 53.3%; Sp: 70%; PPV: 57.1%; NPV: 66.7%
Jabbar S.A. et al., 2020 [29]	*Indian Journal of Forensic Medicine & Toxicology*	Cross-sectional study	Suspicious lesion of oral cavity	50	VELscope	Se: 76.92%; Sp: 64.86%; PPV: 43.48%; NPV: 88.88%
Guida et al., 2021 [30]	*Oral Disease*	Multicentric retrospective study	Oral cavity lesions	84	NBI	NBI IPCLs III–IV for the detection of OSCC: Se: 100%; Sp: 92.6%; PPV: 33.3%; NPV: 100%
Giovannacci et al., 2021 [31]	*Photobiomodulation, Photomedicine, and Laser Surgery*	Retrospective study	OSCC and OPMDs	60	VELscope	Sp: 0.767; Se: 0.810; Acc: 0.788
Nair D. et al., 2021 [32]	*European Archives of Oto-Rhino Laryngology—HN Surgery*	Prospective observational study	Suspicious lesion of oral cavity	50	NBI	Se: 92.67%; Sp: 90.16; PPV: 92.56%; NPV: 91.67%; Acc: 92.16%
Deganello A. et al., 2021 [33]	*The Laryngoscope*	Prospective study	OLP biopsy proven	56	NBI	NBI Se: 100%; Sp: 96%; NPV: 100%; PPV: 71%; Acc: 96%
Ota A. et al., 2022 [34]	*Cancers*	Cross-sectional study	Lesion of oral mucosa suspected for OPMDs/OSCC	60	NBI	Se: 100%; Sp: 80.9%; PPV: 59.1%; NPV: 100%; Acc: 85.0%
Lajolo C. et al., 2022 [35]	*International Journal of Environmental Research and Public Health*	Retrospective study	OPMDs	25	GOCCLES-Tblue	GOCCLES Se: 66%; Sp: 48%; PPV: 34%; NPV: 77%; Acc: 53% Tblue Se: 91%; Sp: 68%; PPV: 55%; NPV:95%; Acc: 75%
Debnath A. et al., 2024 [36]	*Journal of Pharmacy and Bioallied Sciences*	Prospective study	Lesions of oral cavity/oropharynx larynx and hypopharynx	68 patients (33 with oral cavity lesions)	NBI	Se: 100%; Sp: 94.74%; Acc: 98.59

**Table 2:** Overall QUADAS-2 risk of bias assessment.

RISK OF BIAS ASSESSMENT				
N° OF STUDY	PATIENTS’ SELECTION	TEST IN STUDY	REFERENCE STANDARD	FLOW AND TIMING
Study 1 [19]	LOW	LOW	LOW	LOW
Study 2 [20]	LOW	LOW	LOW	LOW
Study 3 [21]	LOW	LOW	LOW	LOW
Study 4 [22]	HIGH	LOW	LOW	LOW
Study 5 [23]	HIGH	LOW	LOW	LOW
Study 6 [24]	LOW	LOW	LOW	LOW
Study 7 [25]	LOW	UNCLEAR	LOW	LOW
Study 8 [26]	LOW	LOW	LOW	LOW
Study 9 [27]	LOW	LOW	LOW	LOW
Study 10 [28]	LOW	LOW	LOW	LOW
Study 11 [29]	HIGH	LOW	LOW	LOW
Study 12 [30]	LOW	LOW	LOW	LOW
Study 13 [31]	UNCLEAR	LOW	LOW	LOW
Study 14 [32]	LOW	LOW	LOW	LOW
Study 15 [33]	LOW	LOW	LOW	LOW
Study 16 [34]	LOW	UNCLEAR	LOW	LOW
Study 17 [35]	LOW	LOW	LOW	LOW
Study 18 [36]	LOW	LOW	LOW	LOW

## Data Availability

No new data were created or analysed in this study. Data sharing is not applicable to this article.

## Supplementary Materials

The following supporting information can be downloaded at https://www.mdpi.com/article/10.3390/jcm15062146/s1, Table S1: PRISMA 2020 Checklist.

## Abbreviations

The following abbreviations are used in this manuscript:

OSCC	Oral squamous cell carcinoma
OPMDs	Oral potentially malignant disorders
OLP	Oral lichen planus
OLL	Oral lichenoid lesion
OLR	Oral lichenoid reaction
ADA	American Dental Association
AAOM	American Academy of Oral Medicine
COE	Conventional oral examination
WL/WLI	White light/white light inspection
NBI	Narrow band imaging
IPCLs	Intrapapillary capillary loops
HGD	High-grade dysplasia
LGD	Low-grade dysplasia
NADH	Nicotinamide Adenine Dinucleotide
FAD	Flavine Adenine Dinucleotide
LAF	Loss of autofluorescence
FVL	Fluorescence visualization loss
FVR	Fluorescence visualization retained
FVI	Fluorescence visualization increase
VELscope	Visually enhanced lesion scope
GOCCLES	Glasses for Oral Cancer Curing Light Exposed Screening
Tblue	Toluidine blue
LOH	Loss of heterozygosis
PPV	Positive predictive value
NPV	Negative predictive value
